# Redescription of *Chribellphoruraallanae* (Christiansen & Bellinger, 1980) (Collembola, Onychiuridae), with comments on the systematic position of the genus

**DOI:** 10.3897/zookeys.803.28265

**Published:** 2018-12-06

**Authors:** Grzegorz Paśnik, Wanda Maria Weiner

**Affiliations:** 1 Institute of Systematic and Evolution of Animals, Polish Academy of Sciences, Sławkowska 17, 31-016 Kraków, Poland Institute of Systematic and Evolution of Animals, Polish Academy of Sciences Kraków Poland

**Keywords:** Morphology, Nearctic, Oligaphorurini, taxonomy

## Abstract

*Chribellphoruraallanae* (Christiansen & Bellinger, 1980), a poorly known Nearctic springtail, is redescribed and important morphological characters are illustrated (Figs 1–7). The genus is characterized by the following characters: postantennal organ with one vesicle divided into five lobes, antennal segment IV with apical vesicle closely flanked by two papillae, sense organ of the third antennal segment with four papilla, four guard setae, two straight sensory clubs and two sensory rods, very similar in shape and length, labial palp of 0-type, abdominal sternum IV divided ventrally into two subsegments, furcal remnant as a finely granulated area with three rows of setae posteriorly, tibiotarsi with clavate setae in distal whorl and anal spines present. The taxonomic status of this *Chribellphorura* is also discussed.

## Introduction

The monotypic genus *Chribellphorura* was established by Weiner (1996) to accommodate Onychiurus (Archaphorura) allanae described by Christiansen and Bellinger (1980) from localities in Oregon, USA. Weiner erected the new genus in the tribe Oligaphorurini and emphased as its distinguishing characters the presence of an apical vesicle on the antennal segment IV and clavate dorso-distal setae on the tibiotarsi.

In connection with a phylogenetic analysis of the Oligaphorurini (Paśnik and Weiner 2017) and in order to clarify the status of *Chribellphorura*, its type species has been reexamined. As the original description of *C.allanae* is incomplete and we have found differences between the original description and the specimen examined, the current paper presents a redescription of the *C.allanae*, illustrations of its most important taxonomic features, and notes on its taxonomic position.

## Material and methods

### Morphological terms

Labial types are named after Fjellberg (1999). Setae on furcal and manubrial areas are notated after Paśnik and Weiner (2017). Setae on anal valves are named following Yosii (1966).

### Abbreviations

**Ant.** antennal segments,

**Th.** thoracic segments,

**Abd.** abdominal segments,

**AIIIO** sensory organ of Ant. III,

**PAO** postantennal organ,

**Ms** s-microsetae (ms) (microsensillum),

**Pso** pseudocellus,

**Psx** parapseudocellus,

**VT** ventral tube.

## Taxonomy

### Chribellphorura allanae

Taxon classificationAnimaliaCollembolaOnychiuridae

(Christiansen & Bellinger, 1980)

Figs 1–6 , 7

Onychiurus (Archaphorura) allanae Christiansen & Bellinger, 1980: 406.

#### Type material.

Paratype, female (No. Insect Collection 529.3270): USA, Corvallis, Benton Co., Oregon, under loose bark of fir tree, 1967, leg. Fitzgerald, housed in Illinois Natural History Survey, Insect Collection.

#### Diagnosis.

Pso formula as 32/122/23343 dorsally and 2/000/00000 ventrally; AIIIO with four papillae, four guard setae, two smooth, straight sensory clubs and two smooth sensory rods in the middle, subequal in length to sensory clubs; Abd. sternum IV divided ventrally into two subsegments; Ant. IV with apical vesicle flanked by two papillae; PAO with one five-lobed vesicle; Labium of type 0; Tibiotarsi I–III with 2,3,3 clavate tenent setae.

#### Redescription.

**Figures 1–6.**
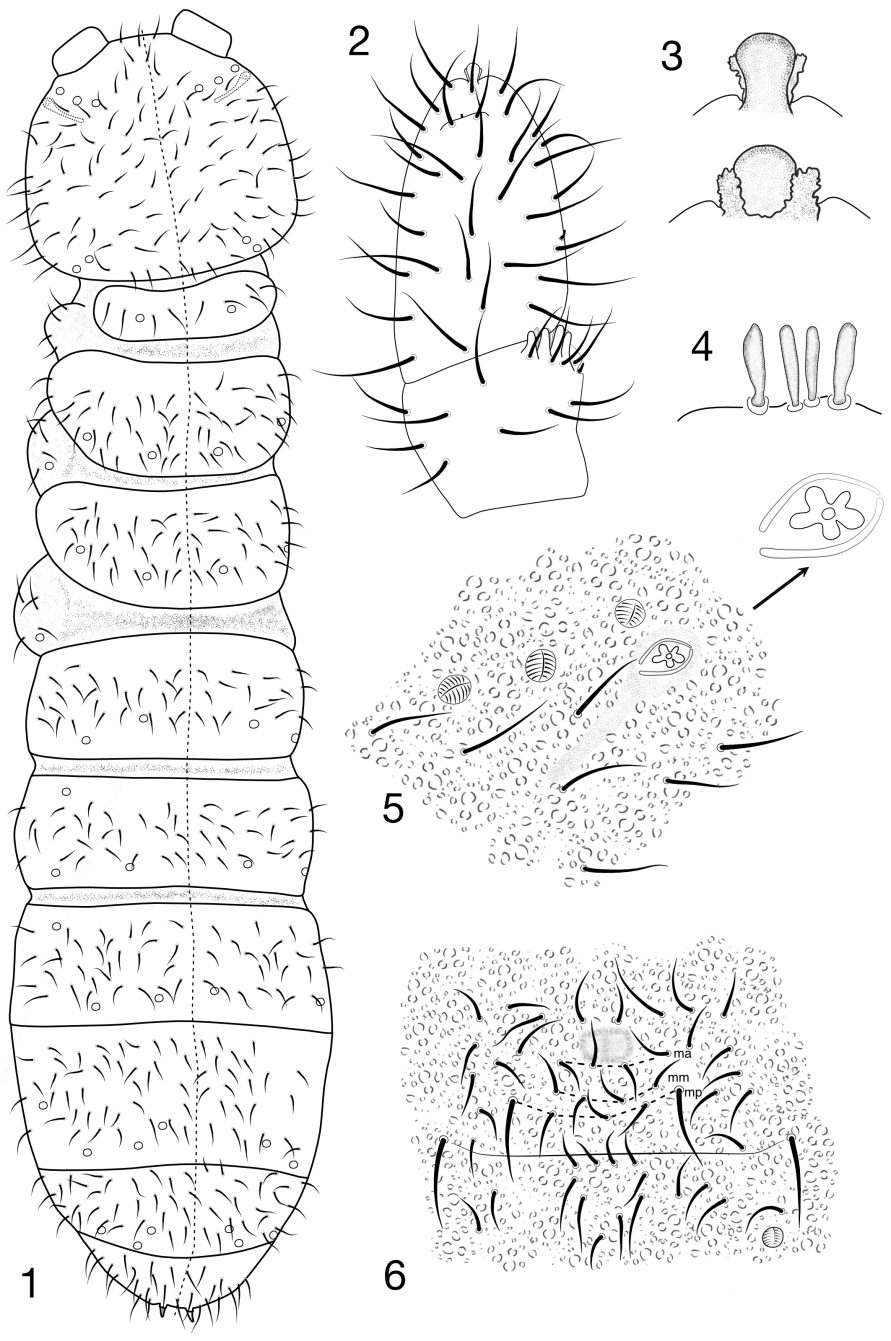
*Chribellphoruraallanae*: **1** Habitus and dorsal chaetotaxy **2** Dorsal side of Ant. III–IV **3**Ant. IV apical vesicle from dorsal (upper) and ventral view (lower) **4** Sensorial elements of AIIIO**5**PAO and anterior cephalic pso **6**Abd. IV, furcal remnant.

Length up to 2.0 mm, specimen examined (female) 1.3 mm. Colour in alcohol white (red when alive; Christiansen and Bellinger 1980). Body shape cylindrical, abdomen slightly broadened in the region of Abd. III and IV (Fig. 1). Granulation homogenous.

Antennae thin, shorter than head, not club-like, their base not marked. Ant. I with 10 setae, Ant. II with 16 setae. AIIIO consisting of four papillae, four guard setae, two smooth, straight sensory clubs and two smooth sensory rods in the middle, subequal in length to sensory clubs (Fig. 4), ventro-lateral s-microsetae (ms) present (Fig. 2). Ant. IV with small subapical organite and apical vesicle with two closely flanked papillae (giving the impression of a trilobed vesicle) (Fig. 3) and s-microsetae (ms) located in the middle of the segment (Fig. 2).

PAO with one five-lobed vesicle (5 or 6 lobed; Christiansen and Bellinger 1980), located in a depression with schitinised edges, subequal in length to the nearest pseudocellus (Fig. 5). Seta d0 on the head absent. Labral formula of setae: 4/342. Maxillary palp simple with two sublobal hairs. Labial type 0. Labium with four basal and five basolateral setae.

Dorsal pso formula as 32/122/23343 (Fig. 1), ventrally as 2/000/00000. Abd. terga II–III with pso c located in antero-lateral position (Fig. 1). Psx indistinct. Subcoxa 1 of legs I–III with 1,1,1 pso and 5,5,5/6 setae respectively. Dorsal chaetotaxy asymmetrical, setae relatively short, weakly differentiated into macro- and microsetae. Sensory setae s undifferentiated. Th. tergum I with 4+5 setae. Th. tergum II–III with lateral s-microsetae (ms). Abd. tergum VI with medial setae a0 and p0. Anal spines small and straight, set on small papillae, 0.33 as long as inner edge of claw (Fig. 1). Th. sterna I–III without setae. VT tube with 7+7 distal setae and 2+2 setae at base.

Abd. sternum IV elongated, divided ventrally into two subsegments, below this division with four setae in the middle of segment (Fig. 6). Furcal vestige reduced to field of fine granulation. Chaetotaxy of manubrial field with four setae in ma-row, four setae in mm-row and five setae in mp-row (Fig. 6). Anal valves with numerous acuminate setae, each lateral valve with a0 and 2a1; upper valves with setae a0, 2b1, 2b2, c0, 2c1, 2c2.

**Figure 7.**
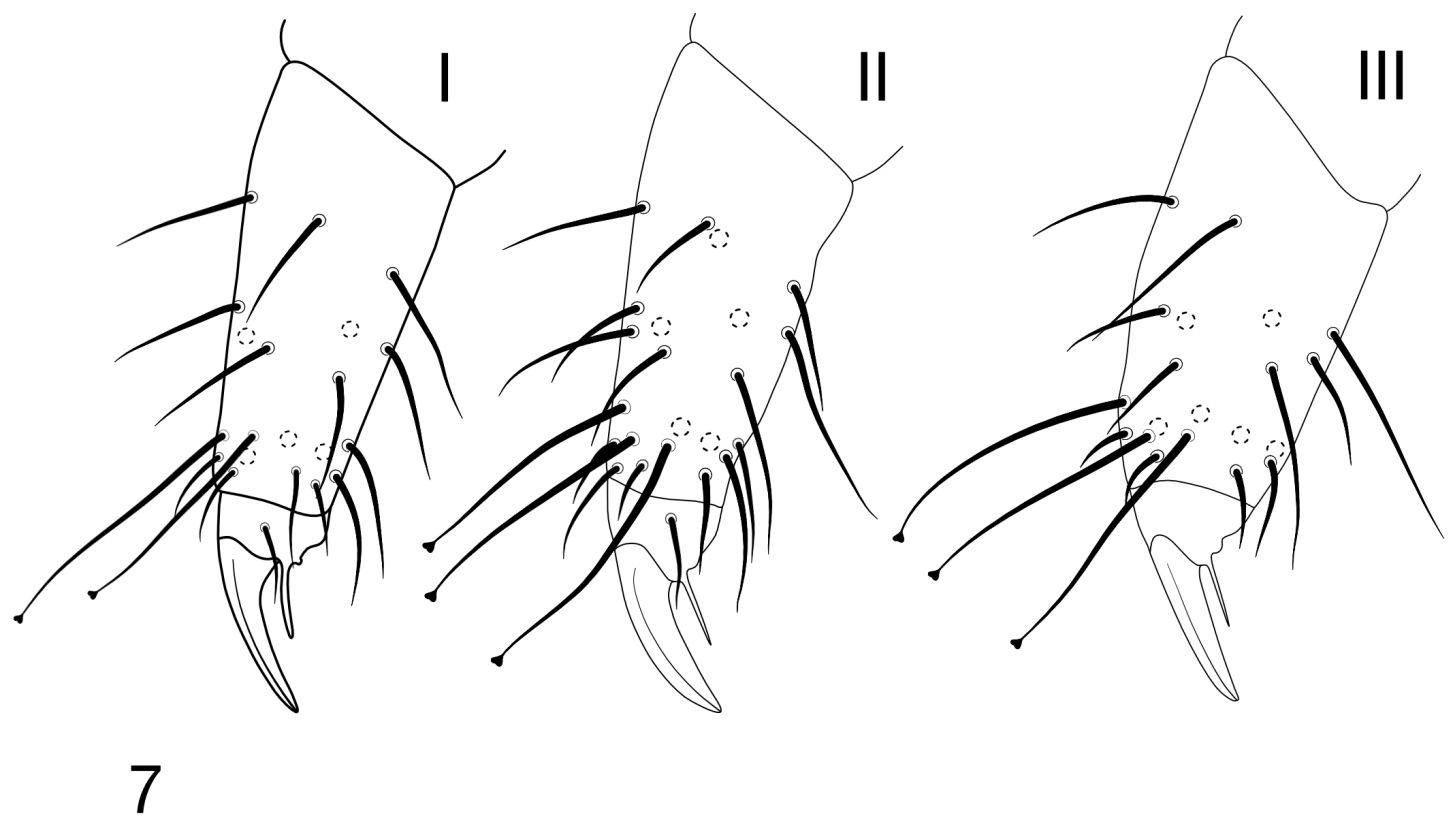
*Chribellphoruraallanae*: Tibiotarsi and claws of legs I–III.

Tibiotarsi I–III with 22, 22, 21 setae respectively, distal tibiotarsal whorl with 11 setae (Fig. 7). Distal whorl on tibiotarsi I–III with 2,3,3 clavate tenent setae respectively (Fig. 7). Claw without denticle. Empodial appendage without basal lamella and 0.45 as long as inner edge of claw (Fig. 7).

**Table 1.** Differences between the original description of *Chribellphoruraallanae* and the studied paratype.

Character	Original description Christiansen and Bellinger (1980)	Paratype studied
Ant. IV – apical vesicle	Trilobed apical bulb	Apical vesicle flanked by two papillae (Fig. 3)
Sensorial elements of AIIIO	three sensory elements shown in fig. 319D (2 clubs and 1 sensory rod?)	two sensory clubs and two sensory rods (Fig. 4)
Th. tergum I – no. of setae	7 setae (fig. 319A)	4+5 setae (Fig. 1)
Th. tergum I – no. of pso	absent (fig. 319A) 1 pso (Table VII)	1 pso
Th. tergum II – no. of pso	3 pso shown (fig. 319A)	2 pso
Th. tergum V – no. of pso	4 pso shown (fig. 319A) 3 pso (Table VII)	3 pso
Abd. sternum IV	no mention	divided into two subsegment
Ventral tube – no. of setae	6-8+6-8 distal setae	7+7 distal and 2+2 basal setae
Anal spines	curved	straight
Tibiotarsi I–III clavate tenent setae in distal whiorl	with 3 strongly clavate tenent hairs	2,3,3 setae present (Fig. 7)
Empodium:Claw ratio	0.60–0.67	0.45

## Discussion

Christiansen and Bellinger’s description of the *Chribellphoruraallanae* is very abbreviated and does not contain features of taxonomic value introduced for this group in later years. In addition, several of the characters in the original description do not match those of the specimen we examined. These differences are shown in Table 1.

Recent phylogenetic studies of Oligaphorurini (Paśnik and Weiner 2017) recover *Chribellphorura* as a monophyletic group. The genus was placed in the basal position on the tree as a sister group to the remaining taxa. This specialised genus has several features which are absent or rare in the subfamily Onychiurinae: antennal segment IV with apical vesicle closely flanked by two papillae; sense organ of the third antennal segment with sensory elements consisting of two sensory clubs and two sensory rods, which are straight and smooth, and very similar in shape and length; labial palp of 0-type and tibiotarsi with clavate setae in distal whorl.

The genus *Chribellphorura* shares only one feature with the other members of Oligaphorurini, namely the shape of the postantennal organ. However, the use of postantennal organ (PAO) as the main feature to divide Onychiurinae into tribes has already been criticized as a feature that often divides related genera into separate evolutionary lines (e.g. Pomorski 1998). In addition, the definition of tribes based on a single character is insufficient.

Traditionally, the tribes of Onychiurinae have previously been defined by character combinations, which mainly include: the shape of the postantennal organ, the build of the sense organ of the third antennal segment, the presence/absence of pseudocelli and anal spines, the reduction of furca, the chaetotaxy of tibiotarsi, and the distribution of sensory setae on the body. Unfortunately, most of these characters are variable, scattered among the genera, and no longer distinguish the different tribes even when used in combination with other features.

At present, the systematic position of *Chribellphorura* within Oligaphorurini is uncertain and should be studied in greater detail.

## Supplementary Material

XML Treatment for Chribellphorura allanae
